# Education to Action: Improving Public Perception of Bats

**DOI:** 10.3390/ani6010006

**Published:** 2016-01-15

**Authors:** Eric Hoffmaster, Jennifer Vonk, Rob Mies

**Affiliations:** 1 Psychology Department, Oakland University, 2200 N. Squirrel Rd., Rochester Hills, MI 48309, USA; elhoffmaster@oakland.edu; 2 Organization for Bat Conservation, Bloomfield Hills, MI 48303, USA; rmies@batconservation.org

**Keywords:** bats, Chiropteran, attitudes, conservation, knowledge, human harm

## Abstract

Public perception of bats has historically been largely negative with bats often portrayed as carriers of disease. Bats are commonly associated with vampire lore and thus elicit largely fearful reactions despite the fact that they are a vital and valuable part of the ecosystem. Bats provide a variety of essential services from pest control to plant pollination. Despite the benefits of bats to the environment and the economy, bats are suffering at the hands of humans. They are victims of turbines, human encroachment, pesticides, and, most recently, white nose syndrome. Because of their critical importance to the environment, humans should do what they can to help protect bats. We propose that humans will be more likely to do so if their perceptions and attitudes toward bats can be significantly improved. In a preliminary study we found some support for the idea that people can be educated about bats through bat oriented events and exhibits, and that this greater knowledge can inspire humans to act to save bats.

## 1. Education to Action: Improving Public Perception of Bats

Bats belong to the Chiropteran order, which is large and diverse—consisting of an estimated 1260 species [1] that vary greatly in their morphology, diet, and behaviors. This diversity has allowed bats to flourish in many ecological niches, ranging from seaside to mountains and deserts. When bats share environments with humans, many benefits, both environmental and economical, are conferred to humans. For example, bats play a significant role in the destruction of pests that destroy human crops, which leads to economic benefits. They also facilitate the pollination of plants that are essential for the health of not just humans, but many other species as well. Their role in the ecosystem is invaluable, and yet is little appreciated. 

Bats are estimated to save corn farmers one billion USD globally by preying on agricultural pests [2]. Additionally, Maine and Boyles [2] found that bats indirectly inhibited fungal growth and toxic compounds harmful to corn through their elimination of these pests. Corn is just one of many types of crop that bats are found to positively impact. In the continental U.S. alone insectivorous bats have been found to be worth more than three billion USD annually due to their destruction of agricultural pests [3]. When one considers that crops like rice feed millions, it becomes clear the enormous benefit of bats to the economy. In 2011 it was reported that 722 million tons was produced globally [4]. Because of the value of this crop, large steps have been taken to limit the damage caused by agricultural pests, which were estimated to cause 10 million tons of rice to be lost in 1991 [5]. Examining the effect that bats had on controlling pests around rice patties, Puig-Montserra *et al.* [6] found that the areas that were least disturbed from agricultural pests were areas where bat boxes had been placed. These bat boxes provide necessary roosts for bats, providing them a home base from which to hunt agricultural pests. Furthermore, the researchers indicated that bat boxes would be more economically viable for farmers to implement compared to the use of pesticides. Puig-Montserra *et al.* [6] indicate that a bat box would cost a rice farmer $28 USD and last at least 10 years with little maintenance, whereas pesticides would cost the farmer $23 USD per year per hectare to use and would need to be re-applied each season. Pesticides in turn, contribute significantly to the loss of bats worldwide.

Bats not only have economic but also ecological value, being important pollinators and seed dispersers. Kunz *et al.* [7] reported that bat pollination occurs in about 528 plant species worldwide, with plant families such as the *Agavaceae* and *Cactaceae* relying significantly on bats for pollination. Kunz *et al.* [7] indicate that one such plant that bats pollinate, *Agave tequilana*, is a critical component of tequila production, which the researchers also point out is a multimillion dollar industry of Mexico.

Bats also assist in the dispersal of plant species such as figs and palms by ingesting and then carrying them to new locations [8]. While the seeds germinate in their digestive tract, bats will often travel a substantial distance before expelling the seeds, allowing them to take root and grow in novel environments. Jamaican fruit bats (*Artibeus jamaicensis*) will consume and then disperse seeds up to 250 m away from the parent tree [9]. Another species of bat, the greater short-nosed fruit bat (*Cynopterus sphinx*), will disperse seeds up to 2 km away [10,11]. Additionally, pollinators have been reported to move pollen from 800 m to 18 km away from the home tree [12,13]. By assisting in pollinating and dispersing seeds, bats play an integral role in maintaining the health and biodiversity of ecosystems. Although the Chiropteran order has been shown to provide significant benefits to humans both economically and environmentally, bats are under threat with numbers of some species drastically declining. The International Union for Conservation of Nature and Natural Resources (IUCN) has categorized known bat species from least concern to extinct. Currently, five species are listed as extinct and 172 species are listed as critically endangered, endangered, or vulnerable [14]. Threats to bats that may impact bat populations include disease, habitat destruction, and the construction of wind turbines.

Although many threats are caused by man, a new natural threat has arisen in the form of white nose syndrome. White nose syndrome (*Pseud-ogymnoascus destructans)* is a fungal disease that plagues bats by growing on their body during the winter while they hibernate [15]. During hibernation, the fungus prematurely awakens the bats causing critical fat reserves to be depleted. Due to the lack of food in the winter, bats are unable to replenish these fat reserves and expire [15]. The disease currently affects at least seven species of hibernating bats and occurs in the mid-Atlantic and northeastern regions of the U.S., as well as two provinces of Canada [16]. Bat numbers in infected regions have been shown to decrease up to 73% [16].

Humans also present multiple threats to bats. One major threat that bats face is from urbanization. As humans encroach on the environment, many bats are displaced from natural foraging areas, which could precipitate a decline in their numbers. Bats in turn may increasingly find homes in human buildings. However, the more bats that roost in buildings, the more likely it is that bats will come into conflict with humans. Bats that do come into contact with humans through roosting in an occupied building are typically evicted due to human fear of bats and the spread of disease. This displacement of bats leads to lower reproductive success and increased mortality [17,18]. Furthermore, many humans harm bats rather than having them removed safely.

Humans have also erected special structures that pose novel challenges to bats. Almost all of the bat surveys conducted around wind turbines have noted the presence of large numbers of dead bats [19]. Bat deaths around wind turbines are thought to number in the hundreds of thousands annually, with estimates ranging from 450,000 to 888,000 in the U.S. [20,21]. Bat species most affected by wind turbines include the Hoary bat (*Lasiurus cinereus*), eastern red bat (*Lasiurus borealis*), and the silver-haired bat (*Lasionycteris noctivagans*) [22]. These bats are migratory species with most deaths occurring in autumn during the bats’ mating/ migration season [23]. The bats may be attracted to the turbines and mistake them as potential roosting sites [24]. Once the bats approach the turbines they are unable to detect the spinning blades due to their speed (300 km/h) and collide with the blades, which leads to severe injury and death [25].

However, although humans pose significant risks to bats, there are also several ways in which the public can assist in saving bats. One such way that the public can assist in saving bats, is by planting wildflower gardens. Wildflower gardens provide a patch of habitat for bats and other wildlife. One benefit to bats is that wildflower gardens also provide a habitat for insects that bats can then prey on. Parkins and Clark [26] found that, in urban areas, buildings that had green roofs (*i.e.*, gardens on the roof) had a higher density of insects and greater amounts of bat activity. Russo and Jones [27] found foraging sites with water to be favored by bats, as they contain large prey densities and provide the bats with an opportunity to drink. Having a place to replenish their water supply is of particular importance as dehydration is a threat to bats, particularly when temperatures are high [28].

Another way the general public can assist with bat conservation is by erecting bat houses on their properties. Flaquer, Torre, and Ruiz-Jarillo [29] reported that 95.6% of the bat boxes they surveyed were occupied during the breeding season. The researchers also state that the number of females with pups in bat houses increased each year during the four years over which their study was conducted. By erecting bat boxes on their properties, the public will be aiding bat conservation, particularly during the breeding season, by providing the bats an alternative roosting site to buildings and homes where they may be harmed by humans or pets. As many homeowners have concerns regarding the diseases that bats may present to humans [30], bat boxes may help ease a home owner’s fear that a bat may decide to roost in their home and come into conflict with them.

Much of the public has a phobia of bats and views them in a negative light. Robins and Regier [31] found that bats, along with other animals such as snakes and mice, were the animals most often affiliated with self-reported phobias. Such phobias could have arisen through misconceptions of these animals or through a bias to see bats as different and hence scary. For example, bats are often thought to be associated with disease [30,31,32,33]. There is some merit to this fear as bats have been linked to diseases such as measles, mumps, and hepatitis C [34,35]. Bats have also been linked to more serious diseases such as SARS-Cov and the Ebola virus [36,37]. Leroy *et al.* [36] found antibodies specific to the Ebola virus in three species of bats. The spread of disease is at least partly due to animal and human conflict. For instance, a 2007 Ebola hemorrhagic fever outbreak in the Democratic Republic of the Congo (DRC) was the result of local villagers hunting migrating fruit bats for bush meat [38]. Subsequently, other factors such as modern transportation and traditional cultural practices where family members must maintain contact with the sick may contribute to the spread of such diseases [39,40]. Although bats are linked with disease, the prevalence with which these diseases affect bat populations may be exaggerated, with rabies being one such example. Contrary to popular belief, rabies is not highly prevalent in bats. Out of 8,262 bats tested, Whitaker and Douglas [32] found only 5.4% of the sample to test positive for rabies. Although many bat species pose a serious risk of disease to humans, estimates are often exaggerated with all bat species generally considered equally dangerous. In one study, it was determined that more than 85% of rabies cases in the U.S. were attributable to a single bat species. The same authors also indicate that rabies exposure from bats is comparable to that from dogs, and less that from cats; however only bats are considered a high-risk vector [41]. Perceptions of risks from bats therefore can sometimes be informed by biases rather than hard evidence.

Another popular misconception is that all bats are considered to drink blood, when, in reality, this is true of only three species [42]. Furthermore, two species of vampire bat feed almost exclusively on wild birds [42], while the common vampire bat (*Desmodus rotundus*) feeds primarily on large livestock and shows little interest in feeding on humans or even pet animals [43]. Vampire bats are also relatively small compared to the cattle that they typically feed upon, and therefore do not consume dangerous amounts of blood in one feeding. In sharp contrast to these facts, Prokop, Fančovičová, and Kubiatko [44] found that 20% of college students surveyed erroneously thought that *most* bats fed on blood. Thus, educating people with regard to these common misconceptions may help reduce negative attitudes towards bats, which may in turn motivate people to behave more charitably toward these important species.

If the public is going to put forth an effort in assisting to save bat populations, they must have a more positive view of bats. As mentioned, this can best be accomplished by educating the public about bats. Kellert *et al.* [45] found that hunters, who are often considered very knowledgeable about large carnivores such as wolves, and who have direct experience with these animals, tended to have more positive views of these animals compared to other members of the public who have less exposure to these animals. Additionally, Prokop *et al.* [44] found that college students who were more knowledgeable about bats also had a more positive view of bats. So how then does one set out to educate the public about bats?

The Organization for Bat Conservation (OBC) located in Bloomfield Hills, MI has made it their mission to educate and inspire the public to save bats. They do so by providing stimulating education programs where the public is able to see live bats, talk to knowledgeable staff, and learn about ways they can help, such as setting up a bat house and planting a wildflower garden. Through their efforts, OBC has increased the number of bat houses sold within the first six months (January–June) annually from 494 bat houses sold in 2013 to 684 bat houses sold in 2015. With regards to donations received from the public there has been an overall increase where the total amount of donations made in the first two quarters of each year more than doubled over a two year period. Website traffic for the organization increased from 121,197 views in 2014 to 224,853 views in 2015 and their Facebook views increased from 9471 views in 2013 to 19,987 views in 2015. Although this is impressive anecdotal data that people have become increasingly interested in bat conservation, the numbers alone do not indicate if the public retains knowledge from these educational programs or if the public would actually be more willing to take part in bat conservation after attending OBC educational programs.

The aim of the current study was to assess people’s knowledge regarding conservation and attitudes toward bats before and after exposure to a bat centered event, and to determine if the educational programs and exhibits at this event influenced people’s retention of this information. Further, we aimed to test whether individuals report themselves as more likely to engage in behaviors that would help save bats after being exposed to the educational programming. It is important to assess the public’s knowledge about bat conservation and their attitudes towards bats after participating in these educational programs to assess the efficacy of such programs to facilitate the organization’s mission to save bats. It is predicted that, after attending an educational conservation program or event, people will be able to report greater knowledge about conservation efforts affecting bats than they were before entering the program, and will also report being more likely to take part in efforts that would assist bat conservation after attending the educational program.

## 2. Methods

### 2.1. Participants 

Forty-seven participants attending the annual Great Lakes Bat Festival (“Batfest”), and 17 visitors to OBC’s bat exhibit “Superhero’s of The Night” on display at the Cranbrook Institute of Science (Bloomfield Hills, MI, USA) in the fall of 2015 consented to complete a survey at the event. Thirty of these individuals (10 men, 20 women) consented to complete the follow up survey (24 from Batfest and 6 from the Superheros of the Night event). Data from these 30 participants was analyzed. Links to the follow-up survey were sent via email three days following the event with an additional reminder one week after the event. All follow-up surveys were completed one week following the event(s). The annual Great Lakes Bat Festival is an event that informs the public about the unique lives of local bat species while also exposing modern myths about bats. The festival also features of number of activities for both adults and children such as arts and crafts or live presentations aimed at educating the public regarding bats. OBC’s bat exhibit “Superheroes of The Night” features live bat enclosures that allow the public to view live bats up close. A staff member or volunteer from OBC is available near exhibits to answer questions and provide additional information. The exhibit also includes interactive displays for children to utilize and learn more about bat conservation. This exhibit was also open during Batfest. All participants had to be at least 18 years old to participate (*M* = 38.54, *SD* = 13.194).

### 2.2. Procedure

Researchers were positioned at the entrance of the bat festival so that they could engage individuals before they entered the festival. Researchers then approached individuals, introduced themselves and stated they were collecting data in collaboration with OBC on the impact of their programs and exhibits. Participants were then told that, in order to assess the impact of OBC’s programs and exhibits, they would also be receiving a post event survey via email after the event. Links to post event surveys were emailed to participants three days after completing the initial survey and participants had approximately ten days to complete the post survey. Participants then completed the consent form and began the survey. Questions assessed participants’ general knowledge of bat conservation and how likely participants were to engage in conservation efforts. Questions consisted of both open-ended and Likert scale questions. The survey consisted of the following questions including information about age and sex.

(a) List as many possible threats to the well-being of bats that you can think of.
(b) List all of the things that you can think of that you can do to help bats.
(c) List all of the ways you can think of that bats are helpful to the environment.
(d) Do you remember how you learned about your answers to the previous questions? If so, can you tell us what you remember about that experience.
(e) How likely are you to do the following things on a scale of 1 (very unlikely) to 5 (very likely);
  - Put up a bat house.
  - Plant a wildflower garden.
  - Donate to a campaign such as “Save the Bats”.
  - Attend another event put on by OBC.
  - Teach your friends about bats.
  - Be more environmentally conscious (reduce pollution, stop littering, consume less palm oil, conserve energy *etc.*).
(f) How do you generally feel about bats?
(g) How scary are bats to you?

First we wished to assess whether attending the Great Lakes Bat festival and walking through the exhibit “Superheroes of The Night”, led to a significant increase in attendees’ knowledge about the threats to the well-being of bats, benefits they provide, and ways that humans can help save them. We counted the number of accurate responses to each of the knowledge questions both pre and post exposure to the event and exhibit. We counted as accurate responses that matched information provided by OBC during the events. For example, threats included human encroachment, wind turbines, pesticides, and white nose syndrome. Benefits of bats included economic, medicinal, seed dispersal, and pest control. Things that could help bats included planting a wildflower garden, putting up bat houses, educating others about bats, donating to save bats and so on. The important second component of our study was to determine whether increases in knowledge and positive regard toward bats, having determined that they occurred, might be associated with increases in propensity to act on behalf of bats.

## 3. Results

Histograms revealed the variables to be normally distributed, so we conducted a series of paired *t*-tests to compare responses. We first tested whether attendance to the festival or exhibit increased visitors’ knowledge about threats to and benefits of bats. Preliminary data indicates a significant increase in knowledge for all questions. Respondents were able to indicate significantly more threats to bats *t* (24) = −2.916, *p* = 0.008, more ways that they could help bats *t* (24) = −5.308, *p* < 0.001, and more ways in which bats benefit the environment *t* (24) = −6.245, *p* < 0.001 after attending the Great Lakes Bat Festival. Respondents also had significantly more positive attitudes regarding bats after attending OBC’s event than they had before attending the event *t* (29) = 2.245, *p* = 0.032. Although the trend was to find bats less scary as well, this finding was not significant. The data appear in Table 1.

**Table 1:** Mean survey responses, with standard deviations in parentheses, pre and post-events.

Question	Pre Score	Post-Score	Significance
Threats	2.60 (1.26)	3.48 (1.30)	0.008
Help	1.52 (1.01)	2.96 (1.17)	0.000
Environment	1.36 (0.76)	2.66 (0.97)	0.000
Bathouse	3.74(1.14)	4.04(1.07)	0.231
Garden	3.39 (1.34)	4.22 (1.09)	0.027
Donate	3.24 (1.27)	3.56 (1.04)	0.188
Attend Event	3.88 (1.24)	4.12 (0.88)	0.298
Teach Others	4.12 (1.05)	4.40 (0.76)	0.271
Environmentally Conscious	4.17 (1.05)	4.50 (0.59)	0.119
Affect toward Bats	4.36 (0.86)	4.64 (0.57)	0.032
Fear of Bats	1.60 (1.04)	1.76 (1.36)	0.461

We next analyzed whether there were significant increases in motivation to take action to help bats following the events. Although we witnessed numerical increases in reported likeliness to engage in all of the activities we surveyed them about, participants were significantly more likely to engage in only one activity; Respondents were significantly more willing to plant a wildflower garden after attending OBC’s event than before *t* (27) = 2.46, *p* = 0.021.

We also wished to know which aspect of the event was most likely to promote increased understanding of bats. Twenty-two participants indicated where and how they learned about bat conservation. Twelve participants indicated that they learned about bats from demonstrations that featured live bats at Bat Fest. In particular they overwhelmingly pointed to the presentation by the director of OBC, Rob Mies, as providing them with increased knowledge about bats. Six participants indicated that they learned about bat conservation from the live bat exhibit (rather than the live animal programs). The remaining four participants indicated that they learned about bat conservation from other sources such as books and wildlife documentaries.

## 4. Discussion

The results of our preliminary survey study indicated that, after attending educational programs such as Batfest, people were more knowledgeable about bats and conservation. People also held more positive attitudes towards bats and were more willing to plant wildflower gardens on their properties in order to help bats. It is important to note that our sample included individuals that were already sufficiently interested in bats to choose to attend the Great Lakes Bat Festival or view the Superheroes of the Night exhibit, and many were already members of OBC, so were already dedicated to supporting bat conservation. This limitation however actually serves to strengthen the impact of our findings. Given that the event was able to significantly increase knowledge in even the most knowledgeable and interested members of the public and increased their positive regard toward bats, we imagine that an even larger impact would be had on members of the public who had little prior knowledge of bat conservation, and potentially more negative attitudes. However, this conjecture awaits further empirical verification.

As this data is preliminary and was obtained from a biased sample, the findings should be interpreted cautiously. Given the very selective and small convenience sample of only 30 participants, further research is needed. Further research is planned that will first increase the sample size by surveying additional visitors to OBC’s Superheroes of the Night exhibit. In addition, further research aims to sample individuals who may not already have an interest in bats. Such individuals may include college students who do not necessarily have an interest in bats, but may come across a presentation that OBC is presenting on their campus, which they decide to attend. These future sampling methods aim at providing a more representative sample of the general public. It is important to determine how educational programs such as those that OBC presents affect those who may not already have a strong interest or positive attitude towards bats because these are the individuals that may not be taking actions to assist in bat conservation, while those who already have a positive attitude toward bats may be already taking action to help bats.

During the Great Lakes Bat festival individuals were able to have access to interactive displays, educated staff, and to view live bats. Being able to view live bats up close and talk with educated staff allows people to have more direct experience with these animals, which may have contributed to their increase in positive attitudes towards bats. This would be in line with past findings indicating that individuals who had more direct experience with a species and who were more knowledgeable of that species had a more positive attitude toward them [44,45]. As live animal demonstrations in this study were reported to be the most memorable source of information about bats, this may also be an indication that direct experience with the animals under safe conditions may play an important role in shaping attitudes toward a particular species.

## 5. Conclusions 

In conclusion, this preliminary study has demonstrated that people are more knowledgeable about bat conservation, have a more positive attitude toward bats, and are more willing to help bats in the form of planting wildflower gardens after attending a conservation educational event or exhibit. However, due to small sample size, and a biased selection of participants, more research is clearly needed to investigate how these educational programs impact people. Additionally, future research should also aim to investigate how these educational programs affect those individuals who may not already have an interest in learning about or helping bats. Lastly, research should aim to investigate how live animal demonstrations impact people’s knowledge of bats and their attitudes regarding bats. Bats are critical to the future of our ecosystems and depend upon human intervention to continue to thrive.
